# Supplementary material for: Dietary Vitamin D Mitigates Coronavirus-Induced Lung Inflammation and Damage in Mice
Source: Viruses. 2023 Dec 15;15(12):2434. doi: 10.3390/v15122434 (PMC10748145; doi:10.3390/v15122434)
Supplement: Supplementary file 1 [file viruses-15-02434-s001.zip › viruses-2742430-supplementary.pdf]

## Supplementary files

### Figures

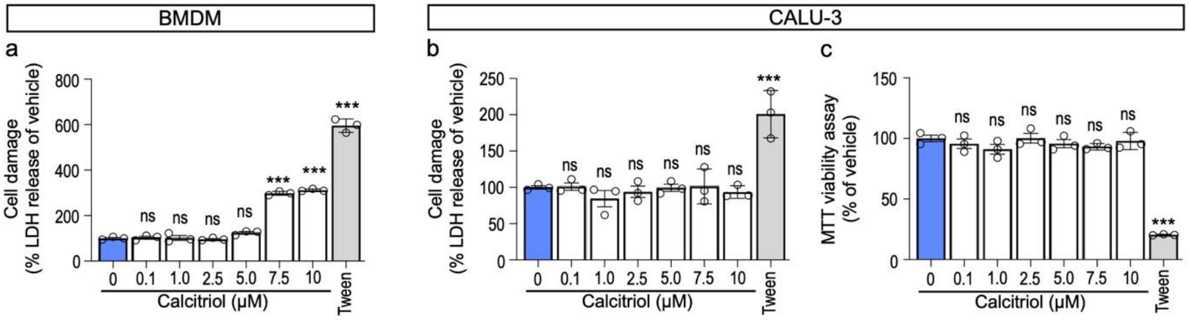


**Figure S1: Calcitriol is cytotoxic to uninfected BMDMs at increasing concentrations, but not to Calu-3 cells.** Calcitriol cytotoxicity was determined by LDH release/activity in the supernatant samples after 24h (a) or 48h (b) of treatment. Additionally, Calu-3 cell viability following calcitriol treatment was assessed by the MTT assay. Differences among vehicle and treated groups were evaluated using one-way ANOVA followed by Dunnett's post-hoc test. ns: non-statistically significant ($p>0.05$). ***$p\leq0.001$. n=3 independent experiments.

## Raw data_Figure 1

| MHV-3 Titer (Log10 p.f.u/mL) - Figure 1b | | | | | |
|---|---|---|---|---|---|
| Replicates | Calcitriol (µM) | | | | |
| | 0 | 0.1 | 1.0 | 2.5 | 5.0 |
| #1 | 7.778 | 7.146 | 7.23 | 7.146 | 5.362 |
| #2 | 7.301 | 7.114 | 7.279 | 6.778 | 5.398 |
| #3 | 7.699 | 7.376 | 6.954 | 7.114 | 5.699 |
| Mean + SEM | 7.593 + 0.15 | 7.212 + 0.08 | 7.154 + 0.10 | 7.013 + 0.12 | 5.486 + 0.11 |
| One-way ANOVA plus Dunnet's (compared to 0 µM) | X | p=0.1146 | p=0.0642 | p=0.0152 | p<0.0001 |

| MHV-3-induced LDH release (% of uninfected control) - Figure 1c | | | | | |
|---|---|---|---|---|---|
| Replicates | Calcitriol (µM) | | | | |
| | 0 | 0.1 | 1.0 | 2.5 | 5.0 |
| #1 | 312.67 | 303.03 | 277.81 | 199.66 | 125.34 |
| #2 | 280.24 | 263.37 | 307.94 | 262.75 | 121.82 |
| #3 | 275.08 | 240.67 | 227.54 | 210.23 | 141.23 |
| Mean + SEM | 289.3 + 11.76 | 269.0 + 18.22 | 271.1 + 23.45 | 224.2 + 19.51 | 129.5 + 5.97 |
| One-way ANOVA plus Dunnet's (compared to 0 µM) | X | 0.8116 | 0.8588 | 0.0664 | 0.0002 |

| MHV-A59 Titer (Log10 p.f.u/mL) - Figure 1d | | | | | |
|---|---|---|---|---|---|
| Replicates | Calcitriol (µM) | | | | |
| | 0 | 0.1 | 1.0 | 2.5 | 5.0 |
| #1 | 7.462 | 7.146 | 7.176 | 7.724 | 6.114 |
| #2 | 6.845 | 7.431 | 7.398 | 7.505 | 5.845 |
| #3 | 7.633 | 7.376 | 7.415 | 7.544 | 6.176 |
| Mean + SEM | 7.313 + 0.24 | 7.318 + 0.15 | 7.330 + 0.08 | 7.591 + 0.07 | 6.045 + 0.10 |
| One-way ANOVA plus Dunnet's (compared to 0 µM) | X | p>0.9999 | p=0.9999 | p=0.4148 | p=0.0002 |

| MHV-A59-induced LDH release (% of uninfected control) - Figure 1e | | | | | |
|---|---|---|---|---|---|
| Replicates | Calcitriol (µM) | | | | |
| | 0 | 0.1 | 1 | 2.5 | 5 |
| #1 | 276.58 | 267.90 | 261.77 | 273.96 | 160.54 |
| #2 | 294.57 | 285.04 | 263.40 | 300.30 | 144.93 |
| #3 | 283.65 | 248.88 | 273.72 | 328.81 | 124.73 |
| Mean + SEM | 284.9 + 5.233 | 267.3 + 10.44 | 266.3 + 3.741 | 301.0 + 15.84 | 143.4 + 10.37 |
| One-way ANOVA plus Dunnet's (compared to 0 µM) | X | 0.5674 | 0.5253 | 0.6372 | <0.0001 |

| SARS-CoV-2 Titer (Log10 p.f.u/mL) - Figure 1f | | | | | | |
|---|---|---|---|---|---|---|
| Replicates | Calcitriol (µM) | | | | | |
| | 0 | 1.0 | 2.5 | 5.0 | 7.5 | 10 |
| #1 | 6.20 | 6.00 | 5.51 | 4.85 | 3.90 | 4.43 |
| #2 | 5.95 | 5.26 | 4.60 | 5.18 | 4.62 | 3.60 |
| #3 | 6.04 | 5.38 | 5.20 | 4.18 | 4.38 | 3.32 |
| Mean + SEM | 6.063 + 0.07 | 5.547 + 0.23 | 5.103 + 0.27 | 4.737 + 0.29 | 4.300 + 0.21 | 3.783 + 0.33 |
| One-way ANOVA plus Dunnet's (compared to 0 µM) | X | p=0.4774 | p=0.0677 | p=0.0108 | p=0.0013 | p=0.0002 |

| SARS-CoV-2 Titer (Log10 p.f.u/mL) - Figure 1g | | | | | | |
|---|---|---|---|---|---|---|
| Replicates | Calcitriol (μM) | | | | | |
| | 0 | 1.0 | 2.5 | 5.0 | 7.5 | 10 |
| #1 | 220.22 | 143.96 | 182.76 | 196.62 | 161.57 | 176.34 |
| #2 | 196.38 | 209.48 | 184.25 | 183.18 | 255.46 | 217.89 |
| #3 | 208.62 | 193.59 | 216.60 | 217.04 | 190.75 | 206.86 |
| Mean ± SEM | 208.4 ± 6.881 | 182.3 ± 19.73 | 194.5 ± 11.04 | 198.9 ± 9.841 | 202.6 ± 27.74 | 200.4 ± 12.43 |
| One-way ANOVA plus Dunnet's (compared to 0 μM) | X | p=0.6884 | p=0.9578 | p=0.9916 | p=0.9987 | p=0.9958 |

# Raw data_Figure 2

**Figure 2b**

| Body weight change (Mean % + S.E.M % from baseline) | | |
|---|---|---|
| dpi | STD_MHV | Vit.D_MHV |
| 0 | 100.0 + 0.000, n=10 | 100.0 + 0.000, n=10 |
| 1 | 99.52 + 1.925, n=10 | 99.21 + 0.488, n=10 |
| 2 | 95.30 + 1.812, n=10 | 97.58 + 1.185, n=10 |
| 3 | 87.64 + 2.467, n=10 | 94.74 + 2.493, n=10 |
| 4 | 82.17 + 2.405, n=10 | 88.61 + 2.553, n=10 |
| 5 | 74.96 + 2.398, n=10 | 82.86 + 2.813, n=9 |
| 6 | 72.00 + 2.000, n=7 | 76.89 + 2.634, n=8 |
| 7 | | 81.44 + 8.370, n=7 |
| 8 | | 82.58 + 6.315, n=4 |
| 9 | | 88.86 + 2.865, n=4 |
| 10 | | 90.61 + 3.370, n=3 |
| 12 | | 93.39 + 1.985, n=3 |
| 14 | | 94.25 + 1.765, n=3 |
| | | |

| Two-way ANOVA n=10 | | | |
|---|---|---|---|
| ANOVA Table | F (DFn, DFd) | | P value |
| dpi (0 to 6 dpi) | F (6, 120) = 1.469 | | P=0.1944 |
| diet factor | F (6, 120) = 47.38 | | P<0.0001 |
| dpi x diet factor | F (1, 120) = 14.02 | | P=0.0003 |
| **Multiple comparisons - Fischer LSD posthoc** | | | |
| STD_MHV - Vit.D_MHV | Sense | FDR | Individual P Value |
| 0 dpi | A-B | 0.750 | >0.9999 |
| 1 dpi | A-B | 0.750 | 0.9114 |
| 2 dpi | A-B | 0.4343 | 0.4137 |
| 3 dpi | A-B | 0.0312 | 0.0119 |
| 4 dpi | A-B | 0.0388 | 0.0222 |
| 5 dpi | A-B | 0.0312 | 0.0066 |
| 6 dpi | A-B | 0.172 | 0.1311 |

**Figure 2c**

| Mean Clinical Score | | |
|---|---|---|
| dpi | STD_MHV | Vit.D_MHV |
| 1 | 0 | 0 |
| 2 | 0 | 0 |
| 3 | 0.8 | 0.2 |
| 4 | 3.2 | 1 |
| 5 | 6.6 | 2 |
| 6 | 11.8 | 3.4 |
| 7 | 15 | 6 |
| 8 | 15 | 7 |
| 9 | 15 | 3.5 |
| 10 | 15 | 0.5 |
| 11 | 15 | 0 |
| 12 | 15 | 0 |
| 13 | 15 | 0 |
| 14 | 15 | 0 |

| Two-way ANOVA n=10 | | |
|---|---|---|
| Fixed effects (type III) | F (DFn, DFd) | P value |
| dpi (all time points) | F (2.242, 10.09) = 96.70 | <0.0001 |
| diet factor | F (1, 8) = 464.6 | <0.0001 |
| dpi x diet factor | F (12, 54) = 48.74 | <0.0001 |

**Figure 2d**

| Survival Analysis | | | | | |
|---|---|---|---|---|---|
| Biological replicate | d.p.i | Mock_STD | Mock_Vit.D | MHV_STD | MHV_Vit.D |
| #1 | 14 | 0 | | | |
| #2 | 14 | 0 | | | |
| #3 | 14 | 0 | | | |
| #4 | 14 | 0 | | | |
| #5 | 14 | 0 | | | |
| | | | | | |
| #1 | 14 | | 0 | | |
| #2 | 14 | | 0 | | |
| #3 | 14 | | 0 | | |
| #4 | 14 | | 0 | | |
| #5 | 14 | | 0 | | |
| | | | | | |
| #1 | 5 | | | 1 | |
| #2 | 5 | | | 1 | |
| #3 | 6 | | | 1 | |
| #4 | 6 | | | 1 | |
| #5 | 6 | | | 1 | |
| #6 | 5 | | | 1 | |
| #7 | 6 | | | 1 | |
| #8 | 6 | | | 1 | |
| #9 | 6 | | | 1 | |
| #10 | 6 | | | 1 | |
| | | | | | |
| #1 | 5 | | | | 1 |
| #2 | 6 | | | | 1 |
| #3 | 14 | | | | 0 |
| #4 | 14 | | | | 0 |
| #5 | 8 | | | | 1 |
| #6 | 7 | | | | 1 |
| #7 | 8 | | | | 1 |
| #8 | 8 | | | | 1 |
| #9 | 10 | | | | 1 |
| #10 | 14 | | | | 0 |
| Legend: 0: survived ; 1: dead. | | | | | |

| Log-rank (Mantel-Cox) test | | |
|---|---|---|
| Median survival | d.p.i | |
| MHV STD | 6 | |
| MHV Vit.D3 | 8 | |
| **Chi square** | **df** | **P value** |
| 10.7 | 1 | 0.0011 |
| Mock groups were not considered in the analysis | | |

**Figure 2e**

| | Body temperature (mean ℃) | | | |
|---|---|---|---|---|
| | STD group | | vit.D group | |
| | Baseline | 3dpi | Baseline | 3dpi |
| | 36.9 | 38.8 | 36.4 | 37.8 |
| | 37.1 | 38.2 | 37.1 | 37.7 |
| | 36.4 | 38.2 | 36.6 | 37.5 |
| | 36.5 | 38.1 | 36.4 | 37.5 |
| | 36.9 | 38 | 37.1 | 37.3 |
| | 36.1 | 37.9 | 36.8 | 37.2 |
| | 36.1 | 37.6 | 36.5 | 37.1 |
| | 36.6 | 37.6 | 36.2 | 37 |
| | 36.3 | 37.5 | 37.2 | 36.9 |
| | 36.5 | 37.4 | 36.9 | 36.7 |
| Mean | 36.54 | 37.93 | 36.72 | 37.27 |
| S.E.M | 0.1077 | 0.1342 | 0.1104 | 0.1126 |

| Paired t test (STD group) | | Paired t test (Vit.D group) | |
|---|---|---|---|
| P value | <0.0001 | P value | 0.0107 |
| t, df | t=11.80, df=9 | t, df | t=3.208, df=9 |
| Number of pairs = 10 | | Number of pairs = 10 | |

**Figure 2f**

| | MHV-3 load in the plasma (Log10 p.f.u / mL) | | | |
|---|---|---|---|---|
| | STD | | Vit.D | |
| | Mock_A | MHV_B | Mock_C | MHV_D |
| | 1.000 | 5.000 | 1.000 | 1.000 |
| | 1.000 | 4.301 | 1.000 | 1.000 |
| | 1.000 | 4.699 | 1.000 | 1.000 |
| | 1.000 | 5.000 | 1.000 | 4.000 |
| | 1.000 | 5.903 | 1.000 | 1.000 |
| | 1.000 | 5.301 | 1.000 | 4.602 |
| | 1.000 | 5.845 | 1.000 | 4.255 |
| | 1.000 | 4.301 | 1.000 | 5.903 |
| Mean | 1.000 | 5.044 | 1.000 | 2.845 |
| S.E.M | 0.000 | 0.219 | 0.000 | 0.724 |

| Mann-Whitney test | | | |
|---|---|---|---|
| Group comparison | Sense | FDR | Individual P Value |
| Mock_STD vs. MHV-3_STD | A-B | ND | ND |
| Mock_Vit.D vs. MHV-3_Vit.D | C-D | ND | ND |
| MHV-3_STD vs. MHV-3_Vit.D | B-D | ND | 0.0162 |
| Mock_STD vs. Mock_Vit.D | A-C | ND | ND |

# Raw data_Figure 3

| Figure 3a | Lung MHV-3 load (Log10 p.f.u / g) | | | | Mann-Whitney test | | | |
|---|---|---|---|---|---|---|---|---|
| | STD | | Vit.D | | Group comparison | Sense | FDR | Individual P Value |
| | Mock_A | MHV_B | Mock_C | MHV_D | Mock_STD vs. MHV-3_STD | A-B | ND | ND |
| | 1.000 | 3.600 | 1.000 | 1.000 | Mock_Vit.D vs. MHV-3_Vit.D | C-D | ND | ND |
| | 1.000 | 4.000 | 1.000 | 3.100 | MHV-3_STD vs. MHV-3_Vit.D | B-D | ND | 0.0079 |
| | 1.000 | 5.000 | 1.000 | 3.000 | Mock_STD vs. Mock_Vit.D | A-C | ND | ND |
| | 1.000 | 4.600 | 1.000 | 1.000 | | | | |
| | 1.000 | 4.100 | 1.000 | 3.000 | | | | |
| Mean | 1.000 | 4.260 | 1.000 | 2.220 | | | | |
| S.E.M | 0.000 | 0.244 | 0.000 | 0.498 | | | | |

Figures 3b and 3c

| Experiment group | Biological Replicate | Cell counts x 10^3/uL blood: WBC | Lymphocytes | Neutrophils | Neutrophil-to-lymphocyte ratio |
|---|---|---|---|---|---|
| Mock_STD | 1 | 4.2 | 2.1 | 2.1 | 1.0 |
| | 2 | 3.7 | 2.4 | 1.3 | 0.5 |
| | 3 | 3.7 | 2 | 1.7 | 0.9 |
| | 4 | 3.5 | 2.1 | 1.4 | 0.7 |
| | 5 | 4.1 | 2.2 | 1.9 | 0.9 |
| Mean | | 3.84 | 2.16 | 1.68 | 0.8 |
| S.E.M | | 0.1327 | 0.06782 | 0.1497 | 0.08944 |
| MHV_STD | 1 | 2.5 | 0.7 | 1.8 | 2.6 |
| | 2 | 2.4 | 0.8 | 1.6 | 2.0 |
| | 3 | 1 | 0.3 | 0.7 | 2.3 |
| | 4 | 1.3 | 0.4 | 0.9 | 2.3 |
| | 5 | 2.4 | 0.6 | 1.8 | 3.0 |
| Mean | | 1.92 | 0.56 | 1.36 | 2.44 |
| S.E.M | | 0.3184 | 0.09274 | 0.2337 | 0.1691 |
| Mock_Vit.D | 1 | 3.1 | 1.9 | 1.2 | 0.6 |
| | 2 | 2.7 | 1.6 | 1.1 | 0.7 |
| | 3 | 4.3 | 2.3 | 2 | 0.9 |
| | 4 | 5 | 3.4 | 1.6 | 0.5 |
| | 5 | 4 | 2 | 2 | 1.0 |
| Mean | | 3.82 | 2.24 | 1.58 | 0.74 |
| S.E.M | | 0.414 | 0.3108 | 0.1908 | 0.09274 |
| MHV_Vit.D | 1 | 3.2 | 1.4 | 1.8 | 1.3 |
| | 2 | 1.4 | 0.6 | 0.8 | 1.3 |
| | 3 | 2.2 | 0.8 | 1.4 | 1.8 |
| | 4 | 3.8 | 1.9 | 1.9 | 1.0 |
| | 5 | 4 | 1.7 | 2.3 | 1.4 |
| Mean | | 2.92 | 1.28 | 1.64 | 1.36 |
| S.E.M | | 0.4923 | 0.2518 | 0.2542 | 0.1288 |

One-way ANOVA plus Fischer LSD posthoc test

Whole blood cells (WBC)

| Group comparison | Sense | FDR | Individual P Value |
|---|---|---|---|
| Mock_STD vs. MHV-3_STD | A-B | 0.0059 | 0.0019 |
| Mock_Vit.D vs. MHV-3_Vit.D | C-D | 0.1054 | 0.1004 |
| MHV-3_STD vs. MHV-3_Vit.D | B-D | 0.1054 | 0.0705 |
| Mock_STD vs. Mock_Vit.D | A-C | 0.7635 | 0.9696 |

Lymphocytes

| Group comparison | Sense | FDR | Individual P Value |
|---|---|---|---|
| Mock_STD vs. MHV-3_STD | A-B | <0.0001 | <0.0001 |
| Mock_Vit.D vs. MHV-3_Vit.D | C-D | 0.0026 | 0.0049 |
| MHV-3_STD vs. MHV-3_Vit.D | B-D | 0.0092 | 0.0263 |
| Mock_STD vs. Mock_Vit.D | A-C | 0.2072 | 0.7892 |

Neutrophils

| Group comparison | Sense | FDR | Individual P Value |
|---|---|---|---|
| Mock_STD vs. MHV-3_STD | A-B | 0.76 | 0.2993 |
| Mock_Vit.D vs. MHV-3_Vit.D | C-D | 0.8853 | 0.8431 |
| MHV-3_STD vs. MHV-3_Vit.D | B-D | 0.76 | 0.3619 |
| Mock_STD vs. Mock_Vit.D | A-C | 0.8853 | 0.7418 |

Neutrophil-to-lymphocyte ratio (NRL)

| Group comparison | Sense | FDR | Individual P Value |
|---|---|---|---|
| Mock_STD vs. MHV-3_STD | A-B | <0.0001 | <0.0001 |
| Mock_Vit.D vs. MHV-3_Vit.D | C-D | 0.0008 | 0.0024 |
| MHV-3_STD vs. MHV-3_Vit.D | B-D | <0.0001 | <0.0001 |
| Mock_STD vs. Mock_Vit.D | A-C | 0.1999 | 0.7616 |

| Figure 3d | CD45+ Leukocytes in Lungs (cells / mm2) | | | | One-way ANOVA plus Fischer LSD posthoc test | | | |
|---|---|---|---|---|---|---|---|---|
| | STD | | Vit.D | | Group comparison | Sense | FDR | Individual P Value |
| | Mock_A | MHV_B | Mock_C | MHV_D | Mock_STD vs. MHV-3_STD | A-B | <0.0001 | <0.0001 |
| | 180 | 1280 | 340 | 560 | Mock_Vit.D vs. MHV-3_Vit.D | C-D | 0.0928 | 0.1326 |
| | 150 | 2310 | 580 | 320 | MHV-3_STD vs. MHV-3_Vit.D | B-D | 0.0007 | 0.0006 |
| | 410 | 1760 | 260 | 1550 | Mock_STD vs. Mock_Vit.D | A-C | 0.5193 | 0.9891 |
| | 180 | 3130 | 330 | 840 | | | | |
| | 700 | 1560 | 130 | 660 | | | | |
| Mean | 324.000 | 2008.000 | 328.000 | 786.000 | | | | |
| S.E.M | 105.000 | 327.300 | 73.310 | 208.700 | | | | |

| Figure 3g | Mean lung injury score | | | | Kruskal-Wallis test plus uncorrected Dunn's test | | | |
|---|---|---|---|---|---|---|---|---|
| | STD | | Vit.D | | Group comparison | Sense | FDR | Individual P Value |
| | Mock_A | MHV_B | Mock_C | MHV_D | Mock_STD vs. MHV-3_STD | A-B | 0.0017 | 0.0006 |
| | 2.000 | 9.000 | 2.000 | 2.000 | Mock_Vit.D vs. MHV-3_Vit.D | C-D | 0.4423 | 0.5617 |
| | 1.000 | 5.000 | 3.000 | 2.000 | MHV-3_STD vs. MHV-3_Vit.D | B-D | 0.0784 | 0.0498 |
| | 1.000 | 7.000 | 2.000 | 3.000 | Mock_STD vs. Mock_Vit.D | A-C | 0.3799 | 0.3618 |
| | 1.000 | 6.000 | 1.000 | 1.000 | | | | |
| | 1.000 | 3.000 | 1.000 | 3.000 | | | | |
| Mean | 1.200 | 6.000 | 1.800 | 2.200 | | | | |
| S.E.M | 0.200 | 1.000 | 0.374 | 0.374 | | | | |

| Figure 3h | Caspase3+ cells in Lungs (cells / mm2) | | | | One-way ANOVA plus Fischer LSD posthoc test | | | |
|---|---|---|---|---|---|---|---|---|
| | STD | | Vit.D | | Group comparison | Sense | FDR | Individual P Value |
| | Mock_A | MHV_B | Mock_C | MHV_D | Mock_STD vs. MHV-3_STD | A-B | <0.0001 | <0.0001 |
| | 20 | 230 | 40 | 70 | Mock_Vit.D vs. MHV-3_Vit.D | C-D | 0.0098 | 0.0281 |
| | 40 | 190 | 60 | 130 | MHV-3_STD vs. MHV-3_Vit.D | B-D | <0.0001 | <0.0001 |
| | 20 | 130 | 20 | 110 | Mock_STD vs. Mock_Vit.D | A-C | 0.1411 | 0.5377 |
| | 50 | 220 | 30 | 80 | | | | |
| | 10 | 150 | 50 | 40 | | | | |
| Mean | 28.000 | 184.000 | 40.000 | 86.000 | | | | |
| S.E.M | 7.348 | 19.390 | 7.071 | 15.680 | | | | |

## Raw data_Figure 4

| Figure 4 | TNF (ng/Lung g-1) | | | | One-way ANOVA plus Fischer LSD posthoc test | | | |
|---|---|---|---|---|---|---|---|---|
| | STD | | Vit.D | | Group comparison | Sense | FDR | Individual P Value |
| | Mock_A | MHV_B | Mock_C | MHV_D | Mock_STD vs. MHV-3_STD | A-B | <0.0001 | <0.0001 |
| | 9.763 | 19.491 | 6.123 | 10.731 | Mock_Vit.D vs. MHV-3_Vit.D | C-D | 0.0093 | 0.0265 |
| | 8.603 | 19.334 | 6.602 | 11.009 | MHV-3_STD vs. MHV-3_Vit.D | B-D | <0.0001 | <0.0001 |
| | 8.292 | 19.928 | 8.267 | 9.535 | Mock_STD vs. Mock_Vit.D | A-C | 0.0615 | 0.2341 |
| | 9.227 | 12.030 | 8.707 | 12.789 | | | | |
| | 9.199 | 18.256 | 7.800 | 8.425 | | | | |
| Mean | 9.017 | 17.810 | 7.500 | 10.500 | | | | |
| S.E.M | 0.258 | 1.470 | 0.492 | 0.734 | | | | |
| | IL-6 (ng/Lung g-1) | | | | One-way ANOVA plus Fischer LSD posthoc test | | | |
| | STD | | Vit.D | | Group comparison | Sense | FDR | Individual P Value |
| | Mock_A | MHV_B | Mock_C | MHV_D | Mock_STD vs. MHV-3_STD | A-B | 0.0003 | 0.0003 |
| | 8.585 | 35.432 | 3.207 | 5.785 | Mock_Vit.D vs. MHV-3_Vit.D | C-D | 0.4087 | 0.7786 |
| | 5.341 | 41.751 | 4.137 | 5.489 | MHV-3_STD vs. MHV-3_Vit.D | B-D | 0.0003 | 0.0003 |
| | 5.267 | 16.689 | 5.816 | 6.239 | Mock_STD vs. Mock_Vit.D | A-C | 0.4087 | 0.7691 |
| | 4.971 | 8.373 | 5.013 | 4.897 | | | | |
| | 5.753 | 41.455 | 4.464 | 7.201 | | | | |
| Mean | 5.983 | 28.740 | 4.527 | 5.922 | | | | |
| S.E.M | 0.662 | 6.840 | 0.436 | 0.387 | | | | |
| | IL-1β (ng/Lung g-1) | | | | One-way ANOVA plus Fischer LSD posthoc test | | | |
| | STD | | Vit.D | | Group comparison | Sense | FDR | Individual P Value |
| | Mock_A | MHV_B | Mock_C | MHV_D | Mock_STD vs. MHV-3_STD | A-B | <0.0001 | <0.0001 |
| | 8.132 | 18.389 | 4.220 | 7.681 | Mock_Vit.D vs. MHV-3_Vit.D | C-D | 0.3484 | 0.4977 |
| | 6.215 | 29.413 | 4.827 | 8.608 | MHV-3_STD vs. MHV-3_Vit.D | B-D | <0.0001 | <0.0001 |
| | 5.948 | 15.632 | 4.312 | 7.958 | Mock_STD vs. Mock_Vit.D | A-C | 0.4211 | 0.8021 |
| | 5.608 | 11.627 | 6.341 | 5.720 | | | | |
| | 3.356 | 29.156 | 6.157 | 5.157 | | | | |
| Mean | 5.852 | 20.840 | 5.171 | 7.025 | | | | |
| S.E.M | 0.763 | 3.610 | 0.453 | 0.671 | | | | |
| | IFN-γ (ng/Lung g-1) | | | | One-way ANOVA plus Fischer LSD posthoc test | | | |
| | STD | | Vit.D | | Group comparison | Sense | FDR | Individual P Value |
| | Mock_A | MHV_B | Mock_C | MHV_D | Mock_STD vs. MHV-3_STD | A-B | <0.0001 | <0.0001 |
| | 2.947 | 18.414 | 0.933 | 3.569 | Mock_Vit.D vs. MHV-3_Vit.D | C-D | 0.2698 | 0.3854 |
| | 2.153 | 17.940 | 1.332 | 4.561 | MHV-3_STD vs. MHV-3_Vit.D | B-D | <0.0001 | <0.0001 |
| | 1.440 | 12.277 | 1.350 | 4.263 | Mock_STD vs. Mock_Vit.D | A-C | 0.456 | 0.8686 |
| | 1.184 | 6.487 | 1.766 | 2.075 | | | | |
| | 1.086 | 25.141 | 1.527 | 2.537 | | | | |
| Mean | 1.762 | 16.050 | 1.381 | 3.401 | | | | |
| S.E.M | 0.350 | 3.142 | 0.137 | 0.481 | | | | |

## Raw data_Figure 5

| Figure 5 | Isg20 | | | | One-way ANOVA plus Fischer LSD posthoc test | | | |
|---|---|---|---|---|---|---|---|---|
| | STD | | Vit.D | | Group comparison | Sense | FDR | Individual P Value |
| | Mock_A | MHV_B | Mock_C | MHV_D | Mock_STD vs. MHV-3_STD | A-B | 0.0213 | 0.0203 |
| | 0.917 | 1.689 | 1.294 | 3.156 | Mock_Vit.D vs. MHV-3_Vit.D | C-D | 0.002 | 0.0009 |
| | 1.297 | 1.347 | 0.936 | 4.548 | MHV-3_STD vs. MHV-3_Vit.D | B-D | 0.0276 | 0.0394 |
| | 1.181 | 3.274 | 1.557 | 3.000 | Mock_STD vs. Mock_Vit.D | A-C | 0.2842 | 0.5414 |
| | 0.604 | 2.627 | 1.373 | 2.501 | | | | |
| Mean FC | 1.000 | 2.234 | 1.290 | 3.301 | | | | |
| S.E.M | 0.154 | 0.440 | 0.130 | 0.439 | | | | |
| | Isg15 | | | | One-way ANOVA plus Fischer LSD posthoc test | | | |
| | STD | | Vit.D | | Group comparison | Sense | FDR | Individual P Value |
| | Mock_A | MHV_B | Mock_C | MHV_D | Mock_STD vs. MHV-3_STD | A-B | 0.4267 | 0.3048 |
| | 0.623 | 1.229 | 0.961 | 1.165 | Mock_Vit.D vs. MHV-3_Vit.D | C-D | 0.0554 | 0.0132 |
| | 1.120 | 1.112 | 1.094 | 1.465 | MHV-3_STD vs. MHV-3_Vit.D | B-D | 0.181 | 0.0862 |
| | 0.950 | 1.227 | 1.238 | 1.528 | Mock_STD vs. Mock_Vit.D | A-C | >0.9999 | 0.972 |
| | 1.305 | 1.058 | 0.726 | 1.563 | | | | |
| Mean FC | 1.000 | 1.157 | 1.005 | 1.430 | | | | |
| S.E.M | 0.145 | 0.043 | 0.109 | 0.091 | | | | |
| | Ifn1b | | | | One-way ANOVA plus Fischer LSD posthoc test | | | |
| | STD | | Vit.D | | Group comparison | Sense | FDR | Individual P Value |
| | Mock_A | MHV_B | Mock_C | MHV_D | Mock_STD vs. MHV-3_STD | A-B | 0.3951 | 0.3763 |
| | 0.992 | 1.104 | 1.112 | 2.369 | Mock_Vit.D vs. MHV-3_Vit.D | C-D | 0.0258 | 0.0082 |
| | 1.097 | 1.546 | 0.779 | 1.441 | MHV-3_STD vs. MHV-3_Vit.D | B-D | 0.0619 | 0.0393 |
| | 1.080 | 1.116 | 1.296 | 1.493 | Mock_STD vs. Mock_Vit.D | A-C | 0.7452 | 0.9463 |
| | 0.831 | 0.998 | 0.870 | 1.386 | | | | |
| Mean FC | 1.000 | 1.191 | 1.014 | 1.672 | | | | |
| S.E.M | 0.061 | 0.121 | 0.117 | 0.233 | | | | |
| | Ceacam 1 | | | | One-way ANOVA plus Fischer LSD posthoc test | | | |
| | STD | | Vit.D | | Group comparison | Sense | FDR | Individual P Value |
| | Mock_A | MHV_B | Mock_C | MHV_D | Mock_STD vs. MHV-3_STD | A-B | 0.6751 | 0.643 |
| | 0.793 | 1.226 | 0.529 | 1.246 | Mock_Vit.D vs. MHV-3_Vit.D | C-D | 0.6751 | 0.5404 |
| | 1.292 | 0.805 | 0.968 | 0.548 | MHV-3_STD vs. MHV-3_Vit.D | B-D | 0.6751 | 0.6307 |
| | 0.714 | 0.963 | 0.878 | 1.066 | Mock_STD vs. Mock_Vit.D | A-C | 0.6751 | 0.5517 |
| | 1.199 | 0.629 | 1.140 | 1.152 | | | | |
| Mean FC | 1.000 | 0.906 | 0.879 | 1.003 | | | | |
| S.E.M | 0.144 | 0.127 | 0.129 | 0.156 | | | | |
| | Def1 | | | | One-way ANOVA plus Fischer LSD posthoc test | | | |
| | STD | | Vit.D | | Group comparison | Sense | FDR | Individual P Value |
| | Mock_A | MHV_B | Mock_C | MHV_D | Mock_STD vs. MHV-3_STD | A-B | 0.9258 | 0.6613 |
| | 0.882 | 1.087 | 1.036 | 1.067 | Mock_Vit.D vs. MHV-3_Vit.D | C-D | 0.9308 | 0.8865 |
| | 1.024 | 0.780 | 1.137 | 1.236 | MHV-3_STD vs. MHV-3_Vit.D | B-D | 0.5633 | 0.2682 |
| | 0.996 | 1.236 | 1.274 | 1.130 | Mock_STD vs. Mock_Vit.D | A-C | 0.4391 | 0.1045 |
| | 1.099 | 1.052 | 1.156 | 1.120 | | | | |
| Mean FC | 1.000 | 1.039 | 1.151 | 1.138 | | | | |
| S.E.M | 0.045 | 0.095 | 0.049 | 0.035 | | | | |

## Raw data_Figure 6

| Figure 3a | Mean Liver injury score | | | | Kruskal-Wallis test plus uncorrected Dunn's test | | | |
|---|---|---|---|---|---|---|---|---|
| | STD | | Vit.D | | Group comparison | Sense | FDR | Individual P Value |
| | Mock_A | MHV_B | Mock_C | MHV_D | Mock_STD vs. MHV-3_STD | A-B | 0.0036 | 0.0017 |
| | 2.000 | 5.000 | 1.000 | 7.000 | Mock_Vit.D vs. MHV-3_Vit.D | C-D | 0.0217 | 0.0206 |
| | 1.000 | 8.000 | 1.000 | 5.000 | MHV-3_STD vs. MHV-3_Vit.D | B-D | 0.1932 | 0.276 |
| | 2.000 | 7.000 | 2.000 | 4.000 | Mock_STD vs. Mock_Vit.D | A-C | 0.4123 | 0.7854 |
| | 1.000 | 7.000 | 2.000 | 4.000 | | | | |
| | 2.000 | 9.000 | 1.000 | 4.000 | | | | |
| Mean | 1.600 | 7.200 | 1.400 | 4.800 | | | | |
| S.E.M | 0.245 | 0.663 | 0.245 | 0.583 | | | | |

| Figure 3d | Mean Liver injury score | | | | Kruskal-Wallis test plus uncorrected Dunn's test | | | |
|---|---|---|---|---|---|---|---|---|
| | STD | | Vit.D | | Group comparison | Sense | FDR | Individual P Value |
| | Mock_A | MHV_B | Mock_C | MHV_D | Mock_STD vs. MHV-3_STD | A-B | 0.0036 | 0.0017 |
| | 2.000 | 5.000 | 1.000 | 7.000 | Mock_Vit.D vs. MHV-3_Vit.D | C-D | 0.0217 | 0.0206 |
| | 1.000 | 8.000 | 1.000 | 5.000 | MHV-3_STD vs. MHV-3_Vit.D | B-D | 0.1932 | 0.276 |
| | 2.000 | 7.000 | 2.000 | 4.000 | Mock_STD vs. Mock_Vit.D | A-C | 0.4123 | 0.7854 |
| | 1.000 | 7.000 | 2.000 | 4.000 | | | | |
| | 2.000 | 9.000 | 1.000 | 4.000 | | | | |
| Mean | 1.600 | 7.200 | 1.400 | 4.800 | | | | |
| S.E.M | 0.245 | 0.663 | 0.245 | 0.583 | | | | |
